# Sustaining optimal performance when the stakes could not be higher: Emotional awareness and resilience in emergency service personnel (with learnings for elite sport)

**DOI:** 10.3389/fpsyg.2022.891585

**Published:** 2022-08-30

**Authors:** Emily Jacobs, Richard J. Keegan

**Affiliations:** ^1^Faculty of Health, University of Canberra, Canberra, ACT, Australia; ^2^Research Institute for Sport and Exercise, Faculty of Health, University of Canberra, Canberra, ACT, Australia

**Keywords:** emergency services, mental health, awareness, coping, elite sport

## Abstract

Emergency services personnel are a high stress occupation, being frequently confronted with highly consequential stressors and expected to perform: without fault; under high pressure; and in unpredictable circumstances. Research often invokes similarities between the experiences of emergency services personnel and elite athletes, opening up the possibility of transferring learnings between these contexts. Both roles involve genuine risks to emotional wellbeing because their occupations involve significant stress. Similarly, both roles face obstacles and injury, and their “success” is dependent on high-quality execution of their skills under pressure. As such, both occupations are required to have resilience and effective coping abilities to ensure psychological well-being. Researchers suggest emotional awareness may be a key variable in the management and maintenance of resilience. This study: (1) explored the experiences of emergency services personnel; (2) characterised connections between emotional awareness and resilience; and (3) reflected on the ways these findings can be extrapolated to elite athletes. We analysed 11 interviews with emergency services personnel. Participants identified resilience as crucial when coping with stress, however, many defined resilience as remaining unaffected by stress rather than, for example, managing and responding to it. Participants defined emotional awareness as understanding their emotions, and they recognised associated benefits for coping, resilience, and burnout. Nevertheless, most participants did not engage in practices to improve their emotional awareness. Barriers, such as maladaptive beliefs and help-seeking stigma, interfered with participants’ ability to cultivate emotional awareness, to promote resilience. In contrast, some participants described profound improvements in resilience and coping following the cultivation of emotional awareness. This finding illustrates that systemic change must target the individual, team, and organisation to correct misperceptions about resilience, emotional awareness, and psychological help-seeking. Developing emotional awareness may help emergency services personnel and other high stress occupations like elite athletes process difficult experiences and enhance their resilience, promoting well-being, and career longevity.

## Introduction

Emergency services personnel–including fire fighters, police officers, ambulance personnel, paramedics, and military personnel—share professional risk factors, including exposure to emotional stress and being responsible for community safety (Haugen et al., 2012). These professionals confront physical and psychological stressors daily, coping with irregular work hours, an unpredictable frequency-and-severity of incidents, and the expectation to perform flawlessly in emergency situations (Smith et al., 2019). Emergency services personnel are at risk of burnout and reduced wellbeing because they regularly face traumatic stressors (Haugen et al., 2012). By comparison, elite athletes are defined as those competing in athletic sport at the professional, Olympic, or collegiate levels (Reardon and Factor, 2010—see Swann et al., 2015 for a more detailed discussion). There are notable similarities between emergency services personnel and elite athletes, where both occupations: (1) are performance-based; (2) experience high expectations, pressure, and stress; and (3) are required to have psychological resilience and effective coping mechanisms (Bicalho and Costa, 2018; Doyle et al., 2021). Furthermore, research outlines that both occupations experience significant mental health concerns including depression, anxiety, burnout, and post-traumatic stress disorder (PTSD), and these conditions directly interfere with their ability to perform and execute their occupational responsibilities (Rice et al., 2016; Regehr and LeBlanc, 2017; Shin and Kong, 2017; Klimley et al., 2018; Aron et al., 2019; Daumiller et al., 2021; Lynch, 2021). For these reasons, the following research: (1) explored the experiences of emergency services personnel; (2) characterised connections between emotional awareness and resilience; and (3) reflected on the ways these findings can be extrapolated to elite athletes. In the following text, therefore, we review our core concepts in relation to both emergency services personnel and elite athletes, such that we may be able to review our findings in relation to both groups.

Resilience and emotional awareness have been discussed as key targets to mitigate emotional ill-health in *both* emergency services personnel and elite athletes (Kinman and Grant, 2011). *Resilience* is defined as the process of flexible coping and adaptation when facing stress (McEwen et al., 2015), and *emotional awareness* is defined as the ability to acknowledge, express, understand, and process emotions (Boden and Thompson, 2015). As emotional awareness involves sensing and processing emotion, related benefits include emotion regulation, effective interpersonal functioning, and a decreased risk of PTSD (Poole et al., 2017). Further, emotion regulation has been proposed to act as a potential resilience mechanism (Zeier et al., 2019). As such, emotion regulation skills help both officers and athletes tolerate negative emotions, to cope with injury, perceived failure, or distressing situations (Berking et al., 2010; Ozdemir, 2019). Nevertheless, to effectively implement emotion regulation skills, one must first have a level of emotional awareness. Evidence shows that emergency services personnel can often struggle to tolerate distress and cope with emotionally confronting situations (Berking et al., 2010). Other research found that athletes with low psychological resilience often had greater mental health concerns, low motivation, and low performance (Ozdemir, 2019). This pattern suggests that emergency services personnel and elite athletes would benefit from cultivating emotional awareness, to build emotion regulation skills and promote resilience to stress. To achieve this goal, we must first understand how these experiences and conceptions—of emotional awareness and resilience—are cultivated, experienced, and navigated by those within the setting.

Researchers suggest that emotional awareness and resilience can be improved—for example through simulation activities, mindfulness, and self-reflection activities (Troy and Mauss, 2011). In sport, in particular, “stress inurement training” and “mental fortitude training” have shown promising impacts on performers’ resilience (Fletcher and Sarkar, 2016; Kegelaers et al., 2021; van Rens et al., 2021; Kent et al., 2022). Several of these studies specifically suggested that emotional awareness, or something synonymous to it, changed in those who benefitted from the training (e.g., Kent et al., 2022). Indeed, Lumley and Schubiner (2019) outlined that resilience and emotional awareness can be learned as skills. In military police personnel, respondents described using emotional awareness as useful in learning from stressful experiences (Crane et al., 2019). Kinman and Grant (2011) found that emotional competencies (e.g., emotional intelligence and reflective ability) explained 47 percent of the variance in resilience. They specified that people with emotional competencies and resilience presented lower perceived stress. While there are now several models describing the benefits of, and approaches for, optimising resilience in athletes (Bryan et al., 2019; den Hartigh et al., 2022; Durand-Bush et al., 2022; Sarkar and Page, 2022)—and these may be potentially transferrable into emergency service settings—there remain uncertainties regarding the implementation of such programs into diverse cultures and context. Hence, there may be transferrable insights back into sport by examining how resilience and its key determinants are enacted in similar, high-stakes high-performance settings. Hence, our study sought to understand whether, and how, emotional awareness may lead to enhanced resilience in both emergency services personnel—with implications for elite sport also explored (Sarrionandia et al., 2018).

Resilience remains a central, if misunderstood concept, as researchers have also determined that resilience is protective against burnout and trauma (Troy and Mauss, 2011). Bensimon (2012) found military personnel with “high resilience” were more likely to experience post-traumatic growth. Similarly, Fletcher (2019) demonstrated that elite athletes with psychological resilience went through a transitional processing following adversity, and developed *adversarial growth* resulting in superior subsequent performance. Gucciardi et al. (2021), working with special forces personnel, emphasised the potential overlap of mental toughness and grit with resilience, and similarities between self-compassion and emotional awareness: again reinforcing the apparent connection between emotional awareness and resilience. Ozdemir (2019) noted that athletes with high psychological resilience often had high subjective well-being, self-esteem, and a high stress threshold. Qualitative research conducted by Lyu et al. (2020) suggested that developing resilience in nurses could alleviate high turnover rates. While research has continued to characterise resilience, popular culture has misconstrued the meaning of resilience: defining it as toughness, emotional control and recovering from difficulties without impact (McGarry et al., 2015). These beliefs have implications for emergency services personnel and elite athletes who are—almost inevitably—faced with high stress, high performance expectation and high adversity (Tatebe et al., 2020; Renkiewicz and Hubble, 2021). For example, some reports have suggested that if negative emotions arise in response to stress, the performer may be appraised as “abnormal” or “weak” (Edwards and Kotera, 2020). As such, the ways that resilience and emotional awareness are understood and culturally enacted in real-world settings is crucial. Paying attention to one’s emotional responses and stress experience—i.e., cultivating emotional awareness—has scope to provide a rich emotional vocabulary, and support the reflection and adaptation following stressful experiences.

One key distinguishing feature of emergency services roles, as opposed to elite sport, is the severe consequences that can eventuate. In the emergency services stress, trauma and PTSD occur when responsibilities do not align with skills or expectations, and the consequences of failing to meet situational demands are severe (Crawford et al., 2010). PTSD is a chronic mental health condition associated with high distress and is triggered by exposure to situations that challenge emotional resources, beliefs, and values (Southwick et al., 2014). Smith et al. (2019) found highly unpredictable work stressors were predictive of burnout, trauma, and PTSD in firefighters. Conversely, PTSD and trauma in elite athletes are often related to direct physical injury, witnessing traumatic events, or abusive dynamics within sports teams (Aron et al., 2019). Qualitative literature exploring trauma in the emergency services describes traumatic events as subjective experiences that shatter assumptions about the world, other people, and themselves (e.g., *“I am safe” vs “the world is not safe”; “I am in control” vs “I am powerless”*—Barratt et al., 2018; Boothroyd et al., 2018). How individuals process the series of emotions that follow a traumatic event (e.g., shock, fear, and sadness) influences the development of trauma responses and PTSD (Garner et al., 2016). Then if maladaptive coping strategies, such as alcohol abuse, are employed, emotional health is at further risk (Nydegger et al., 2011). Arble et al. (2018) reported that firefighters and police officers were prone to employing maladaptive coping strategies and less likely to engage in emotional support when processing stress. Smith et al. (2018) and Ballenger et al. (2010) identified alcohol abuse was frequently used by police officers and firefighters to cope with stress. By comparison, research on the prevalence of trauma-related disorders in elite athletes is limited. Reardon et al. (2019) noted that sport-related injuries and concussions were associated with PTSD in elite athletes. They also found that elite athletes that have experienced trauma may also develop comorbid substance use disorders and utilise substances as a coping mechanism. Similar research by Aron et al. (2019) examining trauma in elite athletes reported strong correlations between PTSD, perfectionism, and compartmentalisation as coping mechanism. However, not all stressful and traumatic experiences trigger adverse psychological consequences, and with effective coping methods and emotional awareness, adaptive change and post-traumatic growth can occur (Kwak and Bae, 2017; Brooks et al., 2018). Fletcher (2019) noted that the combination of psychological (e.g., confidence, awareness, and motivation) and environmental factors (e.g., support and camaraderie) influenced athletes’ ability to adapt, develop resilience and experience growth following stress and adversary. Other research identified that emotional intelligence and awareness predicted self-esteem, self-determination resilience, and growth in semi-professional athletes (Trigueros et al., 2019). As such, successful coping, and resilience in emergency services personnel—forged in the crucible of life-and-death consequences—may carry useful insights to supplement knowledge and strategies in elite sport settings.

There are mental health training programs and support services offered in both the emergency services and in elite athlete programs; however, they are often reported as being insufficient (Barratt et al., 2018; Boothroyd et al., 2018; Reardon et al., 2019). There is a clear necessity to implement a comprehensive framework that emphasises the importance of the prevention and intervention of mental health symptoms in athletes (Purcell et al., 2019). It is therefore essential to broaden the understanding and awareness of PTSD and mental health concerns in both populations, to more effectively address exposure to significant stress and support those affected immediately after traumatic or stressful incident. It is also worth reflecting on how to change systemic organisational cultures that may exacerbate traumatic stress and mental health concerns to create a shift towards building occupational resilience (Barratt et al., 2018; Boothroyd et al., 2018). Both emergency services personnel and elite athletes both face inherent, inescapable challenges and significant pressure. Accumulating research highlights that emotional intelligence and more specifically, emotional awareness may be influential in building psychological resilience in these types of professions (Wagner and Martin, 2012; Fletcher, 2019; Trigueros et al., 2019). Mental health training programs—seeking to promote resilience for frequent, high-stakes and high-performance occupations—may both (a) learn from each other, as argued in the preceding text, and (b) incorporate current best-evidence regarding the “active ingredients” of successful self-management and coping: which appears to include emotional awareness and self-reflection (e.g., Crane et al., 2019).

Emotional awareness is proposed to facilitate adaptation to environmental stressors by informing responses and coping needs, a skill required in the emergency services and elite athletes (Boden and Thompson, 2015). Coholic (2011) found that developing emotional awareness through mindfulness enhanced resilience factors including coping and problem-solving. Williams et al. (2010) found police officers who were mindful and emotionally aware experienced less depression and demonstrated resilience. Similarly, Rajan-Rankin (2014) established acceptance of emotions as integral in the development of resilience. Conversely, low emotional awareness has been linked to difficulty identifying and expressing emotions, maladaptive coping, and burnout (Frewen et al., 2012). Thus, our understanding of how to promote and develop resilient performers would be enhanced by research exploring these meaningful experiences of the emergency services personnel, and how they define resilience and emotional awareness from their own—at times, highly stressful—experiences. Building an understanding of how resilience and emotional awareness are constructed, experienced, and navigated by these personnel may then offer insights into how they can be improved: potentially in several high-performance contexts. As there are notable similarities between emergency services personnel and elite athletes, this research will likely have meaningful insights and implications for the psychological resilience and well-being of elite athletes that can direct future research. In sum, the evidence explored suggests that emotional awareness may be a key variable in enhancing resilience—yet, these high stress occupations still struggle with burnout, and poor mental health outcomes, which further supports our rationale to examine their experiences in more depth. We know very little about what resilience and emotional awareness mean to emergency services personnel, and how they experience these concepts from their perspective in the field. How are resilience, emotional awareness and sustained high-performance enacted and brought to life in this context? The purpose of this qualitative research was to explore the definitions, meaning, and experiences of emotional awareness and resilience in emergency services personnel and to better understand how these concepts relate with one-another in the context of their stressful work environment. We further seek to inform future research by drawing on the meaningful insights from this study in other high stress occupations, such as elite athletes.

## Materials and methods

### Participants

Eleven participants (three females and eight males) from the police, fire-brigade, ambulance, and border patrol services volunteered for this study (see Table 1). Participants ranged in age from 23 to 55 years (*M* = 35.36, SD = 9.67). Exclusion criteria included those: (a) who were seeking psychological services for treatment of stress, psychopathology, or a diagnosed mental disorder; (b) had exited the selected occupations over 12 months ago; or (c) exhibited evidence of pre-existing trauma which impacted their mental health, assessed during a screening survey and prior to commencing the interview (using the K10 scale) (Kessler et al., 2002). The study was approved by the University of Canberra’s Human Research Ethics Committee (HREC-2018/1579).

**Table 1:** Participant demographics.

Identifier	Occupation	Sex	Age	Work experience
F&R_1	Fire & Rescue	Male	31	2 years 8 months
F&R_2	Fire & Rescue	Male	32	2 years 10 months
F&R_3	Fire & Rescue	Male	45	11 years 0 months
F&R_4	Fire & Rescue	Female	34	3 years 0 months
AMB_5	Ambulance Service	Female	55	6 years 10 months
AMB_6	Ambulance Service	Male	48	10 years 1 month
AFP_7	Australian Federal Police	Male	29	5 years 7 months
AFP_8	Australian Federal Police	Male	23	1 year 1 month
AFP_9	Australian Federal Police	Male	29	1 year 1 month
AFP_10	Australian Federal Police	Male	31	5 years 3 months
ABF_11	Australian Border Force	Female	32	7 years 0 months

### Procedures

The researchers distributed information sheets outlining the study procedure, benefits, and risks of participation in public areas in the Australian Capital Territory (ACT), through personal networks and social media. Participants voluntarily contacted the researchers if they were interested in participating and provided informed consent. We administered a screening protocol to ensure participants’ appropriateness for inclusion and to collect demographic information.

#### Interview questions

Each participant completed one 30–60 min (*M =* 38.70, SD = 9.64) semi-structured interview to explore their experiences. The interview guide contained questions and probes about their experiences of emotional awareness, resilience, coping with stress, burnout, and help-seeking (see Table 2). The interview guide was developed based on a review of the literature, discussions with experienced qualitative researchers, and examination of other similar studies that utilised open-ended interview styles to ensure participants were able to discuss areas of their work that they felt were important, and to specifically cover the breadth of emotional awareness and resilience. To ensure data collection consistency, the researchers engaged in role play practice sessions.

**Table 2:** The semi-structured interview.

Proposed Interview Questions
What does emotional awareness mean to you?
What does resilience mean to you?
What aspect of your job or situations you come across in your work require resilience?
Tell me about your parents and your childhood home life
How did your family talk about emotions/feelings?
Tell me about an experience at work that was highly stressful for you, and how did you cope?
Have you engaged in any practice to improve your coping?
Have you engaged in any practice to improve your emotional awareness?
Have you felt comfortable seeking a support services through your work or personally?
What are you experiences with burnout?
Tell me about your coping strategies? (Both positive and negative)

### Analysis

For qualitative research to be rigorous and trustworthy, it must have clear epistemological underpinnings (McGannon et al., 2019). The methodology of this study was guided by the recommendations adapted from constructivism and thematic analysis (Braun and Clarke, 2006; Sparkes and Smith, 2009, 2014; Peck and Mummery, 2018). Constructivism acknowledges the existence of multiple social realities and shared meaning and recognises that knowledge is a creation influenced by both the researchers and participants (Smith and McGannon, 2018). Consistent with this epistemology, our goals were to identify themes (and relationships between themes) to create one or more stories, while representing the subjective reality influenced by the interaction between participants and researcher in a trustworthy and authentic process (Braun et al., 2018). As such, the participants’ shared meaning and interpretation of concepts based on their experiences are the data that formed themes. Thematic analysis is a method for identifying, analysing, and reporting patterns (themes) within data (Braun and Clarke, 2006). The themes capture important patterned information about the date in relation to the research question. We followed the methods of thematic analysis (Braun and Clarke, 2006; Smith and Sparkes, 2009; Vaismoradi et al., 2016) by enacting the following transparent process: we conducted interviews in person, then audio recorded, transcribed, and analysed the interviews for codes (meaningful statements), sub-themes, and themes. We did not use any software for the analysis or coding procedures (Guest et al., 2011). Due to practical and time constrains, we were unable to transcribe and analyse each interview before undertaking the next, but the lead researcher did reflect in depth between interviews: using this to inform recruitment decisions and focus areas for subsequent interviews. For transparency, it is beneficial to further explain how we reconciled philosophical considerations, practical constraints, and our own history as researchers. Guided by Caron et al. (2022), we acknowledged that our personal and professional experiences—in sport and performance psychology and clinical psychology—would likely influence our research perspectives and the co-construction of knowledge in this study. The lead female researcher was a clinical psychologist providing advanced trauma-informed clinical interventions to psychiatric inpatients with complex presentations including PTSD. The second male researcher was a registered psychologist working in sport, exercise, and performance psychology, with extensive research and applied experience spanning elite sport, youth sport, and the military. Understanding and constantly, reflecting on our on backgrounds informed our own interpretation of data, and may help the reader to make an appraisal of both the way themes were developed, and ultimately the credibility to the findings.

The lead researcher audio recorded the interviews using a Dictaphone, and then transcribed them verbatim. We reviewed each interview and then conducted coding and thematic analysis iteratively to ensure themes were informed by the development of codes over time. We enacted coding to identify key sections within the text (e.g., a code represents a meaningful quote or statement) and to develop labels representing the meaning of these sections. We grouped codes into categories of conceptually related data and identified the preliminary concepts. Throughout this process, we used continuous comparison to compare codes and categories within and across interviews to establish sub-themes and themes and how they interrelated (e.g., participants held differing views on what resilience represented, and we took time to reflect on how, why, and in which ways participants differed on their description of resilience). The stages of analysis included: closely reading and converting segments of interview data into codes; noting interpretations, connections, and patterns as they became evident to the researchers; identifying patterns that formed sub-themes and themes, continuous reflection of processes underpinning participant experiences; developing a structure to organise codes, sub-themes, and themes; interpreting and explaining themes to form results; double hermeneutics achieved by reflection on the researcher’s perceptions of participant claims (Vaismoradi et al., 2016). We then labelled each theme and sub-theme with explicit descriptions that captured the meaning. Finally, we transformed the data into a written report, supported by empirical evidence. While the analysis of data focused on the first two aims—(1) exploring the experiences of emergency services personnel; and (2) characterised connections between emotional awareness and resilience—we subsequently reflected on the ways these findings can be extrapolated to elite athletes (i.e., Aim 3).

In contrast to the checklist approach to trustworthiness encouraged by realist researchers, adopting a social constructivist approach designates that the evaluation of rigor should be adapted in the context of the ongoing study (Connelly, 2016). As such, our primary rigor-enhancing strategies were: obtaining member reflections on the findings (Smith and McGannon, 2018) to generate additional insights and perspectives, create an intellectually enriched understanding of the findings, explore gaps in the results and to ensure the findings were presented in an ethical, sensitive and respectful manner; and critique from independent colleagues encouraging critical dialogue and reflexivity to challenge the researchers’ construction knowledge (Smith and McGannon, 2018). We conducted and recorded these rigor-enhancing steps through transcribed conversations with participants and colleagues and through email correspondence. We also sought participants from varying positions in emergency services to derive varying personal perspectives, to recognise that each service will carry unique interpretations and biases.

In our judgement, we achieved strong representation of the themes and theoretical insights from the narratives. We attained partial inductive thematic saturation whereby minimal new codes and themes were gained from the data. This realisation indicated that data collection and analysis could be concluded, and the collected data were meaningful (Morse, 1995; O'Reilly and Parker, 2013; Saunders et al., 2018).

## Results

**Figure 1:**
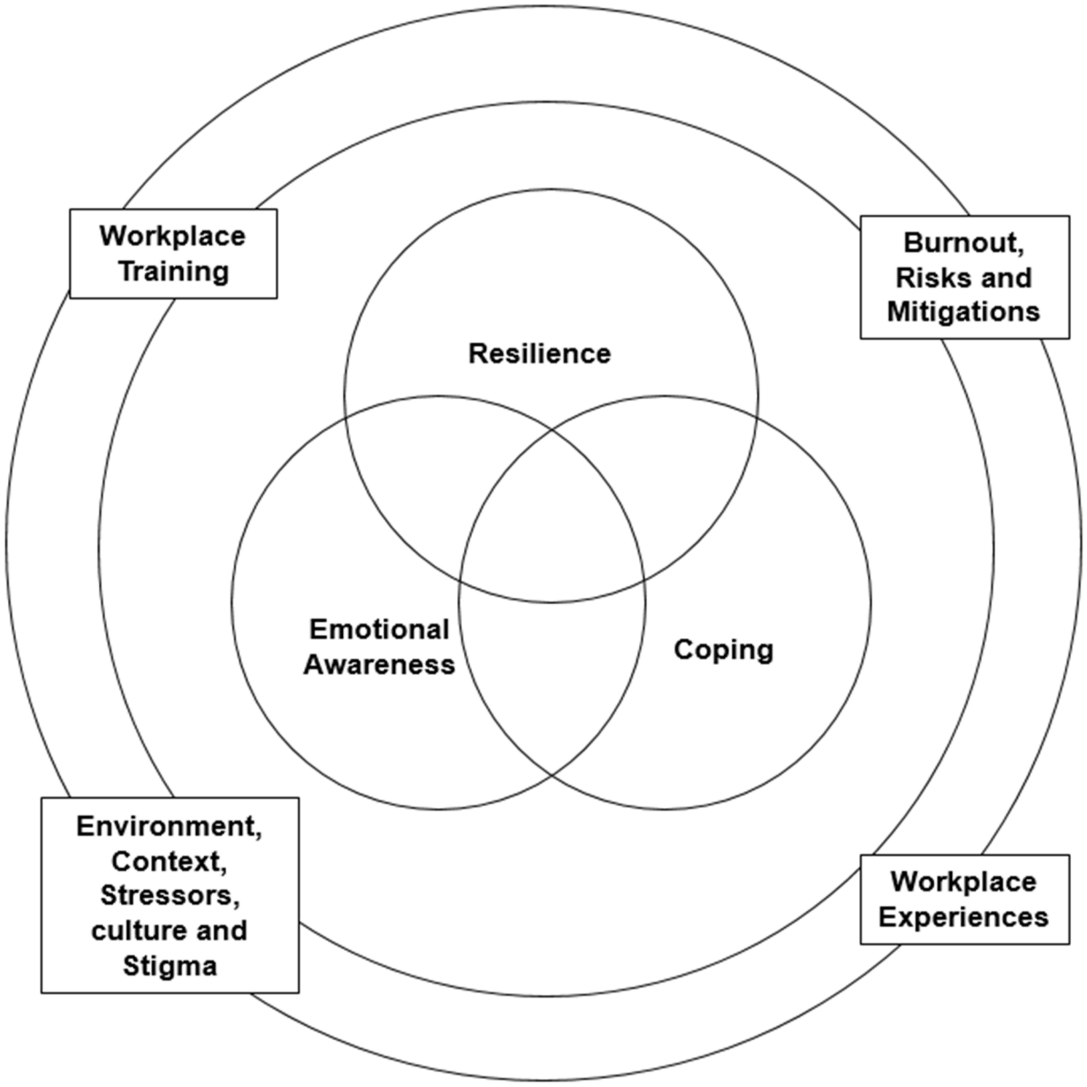
This framework illustrates the connection between resilience, emotional awareness, and coping in the context of the emergency services as a high stress occupation. The coping ability and level of emotional awareness and resilience an individual has impacts whether these constructs are protective factors when facing occupational experiences and stress. Regarding Aim 2—to characterise relationships, our interpretive position upon completion of this analysis was that it was best to represent these phenomena in a Venn diagram—richly connected and interdependent—and not in the form of boxes with arrow suggesting one-way “causality.”

The results are organised into themes and sub-themes that we developed from the data to illustrate the experiences of the emergency services personnel. Supporting quotes from participants are presented in the text throughout the results section. Participants are identified by number and occupation (e.g., fire-and-rescue indicated by F&R_1). The seven interlinked themes were: (1) historical experiences; (2) environment and context; (3) resilience; (4) emotional awareness; (5) coping; (6) workplace experiences; and (7) burnout. In this section we present each overarching theme, followed by its associated sub-themes. Table 3 illustrates the naming convention that structures the accompanying text narrative: Themes (heading, definition, and meaning); and sub-themes (definition and meaning). Figure 1 illustrates the relationship of the key themes.

**Table 3:** Naming convention for themes and sub-themes.

Theme	Sub-Theme
Historical Experiences	(a) Childhood Experiences
	(b) Emotional Expression
Environment and Context	(a) Workplace Training
	(b) Environmental Stressors
	(c) Workplace Culture and Stigma
	(d) Unpredictability and Control
Resilience	(a) Bounce Back
	(b) Just Keep Going
	(c) Managing Distressed People
Emotional Awareness	(a) Understanding Emotions, Triggers, and Reactions
	(b) Emotional Awareness and Communication
	(c) Emotional Awareness Informs Coping and Resilience
Coping	(a) Adaptive Coping
	(b) Maladaptive Coping
Workplace Experiences	(a) Positive Experiences
	(b) Traumatic Experiences
Burnout	(a) Risks and Mitigations of Burnout
	(b) Bucket of Bad Experiences

### Historical experiences

The researchers organised the theme *Historical Experiences* into two sub-themes: (a) Childhood Experiences and (b) Emotional Expression. This theme provides insight into the predispositions and childhood experiences of participants, which is known to impact resilience, emotional functioning and coping mechanisms in adulthood and therefore was deemed important to explore (Zetino et al., 2020). Participants outlined that the nature of their childhood experiences (e.g., emotional connectedness, emotional deprivation, and traumatic experiences) impacted the development of their ability to cope, express emotions, and learn to be interpersonally effective. For example, participant AFP_10 reported: “We had a loving family life and childhood,” reflecting emotional connectedness and support. By contrast, F&R_2 stated: “I just felt hopeless knowing that there’s going to be repercussions for my actions, and they are going to be really bad”—suggesting fear and helplessness, both of which are predictors of depression and post-traumatic stress (Salcioglu et al., 2017). Four of 11 participants described openly discussing their emotions with family and friends, indicating emotional awareness and expression. For example, AMB_6 reported: “There was a lot of inquiry into how people were feeling.” In contrast, F&R_2 stated, “Most of the time I would just keep it inside”—suggesting emotional inhibition. This theme points to the importance of historical experiences—i.e., previous life and occupational experiences that indoctrinate one into a profession—as predispositions in subsequent coping behaviours of participants. We reflected that this theme would likely apply in elite sport quite similarly, although the two subthemes may be more closely entwined as athletes usually engage in sport from an early age.

### Environment and context

We derived the theme *Environment and Context* from participants’ statements about the stressful nature of the work environment, with four sub-themes: (a) Workplace Training; (b) Environmental Stressors; (c) Workplace Culture and Stigma; and (d) Unpredictability and Control.

#### Workplace training

We explored workplace training participants had completed as training and perceived preparedness are helpful when coping with stressful situations (Abbasi et al., 2010). While some participants referred to having engaged in workplace training on psychological resilience and mental health, many described feeling underprepared during stressful incidents, with references made to their ‘first job’: believing more comprehensive training would have further developed their self-efficacy. For example, FandR_1 stated, “So not a fire alarm or anything simple like that, the first working job I went to was to perform CPR for a heroin overdose,” suggesting he perceived this as stressful. It was also noted by AFP_9 that the mental health training received is often dismissed: e.g., “A lot of training is dismissed because they see them [the trainers] as civilian people trying to put themselves in the shoes of cops, the whole cop versus civilian mentality, a lot of the older guys have that, which is disappointing.” This theme suggests that although emergency services personnel do receive training, there are possible barriers interfering with their receptiveness: in this case a belief that there is a categorical difference between emergency services work and many other workplaces, rendering any training program developed outside emergency services irrelevant.

#### Environmental stressors

We formed this sub-theme by collecting statements about stressful environmental experiences including exposure to dead bodies, suicides, traumatic accidents, distressed individuals, significant danger, and uncomfortable environments such as extreme heat and small spaces. To illustrate this, F&R_1 reported, “One of the firie [firefighter] guys on my shift had a CPR job resulting in death every day of that set, including a 3-month-old baby.” Relatedly, AFP_7 stated, “You remove yourself from the situation and you just go, it’s a dead body, and you do not want to get caught up with it, or be emotional about it, grossed out by it, and you just accept it for what it is.”

#### Workplace culture and stigma

Seven male participants reported not feeling comfortable seeking professional help through work, whereas the three female participants did. For example, F&R_3 stated, “I have never [sought a support service], whether that’s out of being uncomfortable with it or a generational thing” and F&R_2 reported, “I’m a dude, its fine, I’ll just deal with it, it’s not that bad,” whereas F&R_4 reflected, “I’ve seen a psychologist before, it was great, I just needed to go and talk, I instantly felt better, it’s like training for your mind.” Participants disclosed that help-seeking stigma and beliefs about masculinity, fears of being pulled out of the field, or appearing incompetent to their colleagues inhibited them from discussing distressing experiences (e.g., AFP_7 stated, “I’m hesitating, it’s the stigma that they’ll pull you off road or it will go on your record”). Although some participants acknowledged a need for psychological services, they were wary of potential professional implications. When we explored how organisations could reduce mental health stigma and mitigate related adverse consequences such as suicide, unhelpful systemic beliefs were apparent, e.g., AFP_9 reported, “At the end of the day if someone [an officer] is going to go and neck themselves, they are going to do it, that’s just how it is.”

#### Unpredictability and control

Participants spoke about unpredictable stressful jobs, and this created on-going hyperarousal. Participants described needing to have acceptance and resilience in response to adverse outcomes, despite their efforts to improve situations. Participants with an internal locus of control reported correctly appraising adverse outcomes without self-blaming and how this process required resilience. For example, AMB_6 reported, “Having an understanding that being resilient towards not being able to fix everything.” This statement reflects a level of awareness about the importance of psychological flexibility and resilience in response to difficult situations.

Overall, we reflected that the theme *Environment and Context* would be likely to have strong parallels to elite sport, although the nature of the environmental stressors seemed likely to differ in important ways (e.g., fewer life-and-death scenarios). Workplace training would not hold the same meaning in sport, but seemed reminiscent of the specific trainings athletes receive regarding media interaction, doping, or anti-corruption initiatives. The acceptance of uncontrollable events seems well discussed in elite sport settings, and similarly to potential for stigma and negative attitudes towards help-seeking appeared similarly possible.

### Resilience

The researchers focussed on *Resilience* as a central theme to explore the diverse experiences of the emergency services personnel and to gain an understanding of how they defined resilience. Participants’ definitions of resilience varied in meaning, so we derived three sub-themes for clarity: (a) Bounce Back; (b) Just Keep Going; and (c) Managing Distressed People. The term “bounce back” was used by three participants to define resilience as the ability to bounce back from visually disturbing and stressful scenes, implementing coping measure, or returning to a previous state of functioning. For example, F&R_2 outlined, “To be able to bounce back from seeing nasty things, you have measures to put in place to deal with that and be able to bounce back and do it again the next day” whereas, AMB_6 stated, “The capacity to bounce back, to return to a previous state after experiencing trials or trauma.” The most salient definition of resilience made by participants was having the ability to remain unemotional and unaffected by difficult experiences, to withstand stress and not get broken down. This meaning was further explained by participant AFP_7 who stated, “It’s just your ability to overcome stuff and not get emotional about it” and AFP_9 outlined, “To keep going, not get broken down and to be able to control your own emotions.” This understanding differed from literature defining resilience as adapting to difficult experiences through flexible coping (Woods, 2017). Participants also stated they needed resilience when interacting with distressed people, for example, F&R_1 described, “You need resilience when dealing with people—not just people that are hurt but people that emotionally distressed which is sometimes a lot harder.” Resilience was interlinked with other themes, for example, participants reported needing resilience for the unpredictability of their work (Environment and Context Theme) and when coping themselves (Coping Theme).

Following this focus on the data, we reflected that the way participants characterised resilience bore many similarities to sport when we considered “bounce-back” and “just keep going”—noting that in fact other ways of conceiving resilience were omitted, such as being highly flexible, or adaptable to novel challenges. The theme of “managing distressed people” seemed relatively novel on first consideration, but of course in team sports—and the above-described models of team resilience—such a theme may well be likely to manifest and be crucial to the performance outcomes of a sporting team. Calming down distressed team-mates, or re-energising them after a setback, may be sporting analogues to this theme as reported by emergency services personnel.

### Emotional awareness

As researchers we also focussed on *Emotional Awareness* as a key theme, exploring how the emergency services personnel defined emotional awareness and the role of emotional awareness in their work. The meaning of emotional awareness was diverse, so we structured three sub-themes: (a) Understanding Emotions, Triggers and Reactions; (b) Emotional Awareness and Communication; and (c) Emotional Awareness Informs Coping and Resilience. When asked about emotional awareness, participants distinguished between interpersonal emotional awareness and intrapersonal activities relating to emotional awareness, both important facets of emotional intelligence (Maraichelvi and Rajan, 2013; Praditsang et al., 2015). All 11 participants defined emotional awareness as having awareness of and understanding their own emotions and emotional reactions (intrapersonal emotional awareness). AFP_9 stated, “An understanding of your own emotions” and ABF_11 reflected, “It means to be self-aware, and aware of how you react within certain situations.” Participants described needing emotional awareness to work empathetically with distressed people, to read other people’s emotions and communicate effectively (interpersonal emotional awareness). For example, five participants reported that emotional awareness helped their ability to notice emotions in other people (e.g., AFP_7 stated, “Being able to recognise how other people feel”); six participants described needing emotional awareness to help support and communicate with distressed people (e.g., F&R_3 reported, “If I turn up to an accident and people are quite distressed, I cannot just walk in and do my job, I have to show a bit of empathy”). Nine participants reflected that emotional awareness helped recognise when to implement effective coping strategies (e.g., F&R_1 stated, “If you are looking at managing traumatic incidents, having an awareness of when things might be getting too stressful—might prompt you to go—maybe I need some help, or maybe I need to use some of my own coping strategies”). One participant reflected the importance of training in resilience and emotional awareness to mitigate risks of emotional ill-health and suicide. For example:

Our department offers resilience training, but personally I do not think that’s sufficient for what some of the officers deal with. They’re not prepped for what they might encounter, so, even though we have kind of ticked boxes, it’s not enough to sustain them in their career and emotional awareness in that respect is super important, because there is actually a high suicide rate in our officers (ABF_1).

Participants provided definitions and insights into the utility of emotional awareness; however, most participants were unaware of how to cultivate or enhance it. Only four participants engaged in practices, such as self-reflection, to improve their emotional awareness. On subsequent reflection, we would position *Emotional Awareness* as likely to be similarly construed and experienced in elite sport: something potentially useful and thus promising, but also incompletely understood and thus underutilised.

### Coping

We organised the theme *Coping* into two sub-themes exploring coping mechanisms reported by participants, labelled: (a) Adaptive Coping, and (b) Maladaptive Coping (Supplementary Table 1). Adaptive coping strategies were behaviours that managed stress, promoted mental health and resilience included mindfulness, social support, reflection, and time off (Wagner and Martin, 2012). Maladaptive coping such as avoidance, poor self-care, and drug and alcohol use temporarily relieved stress; however, these strategies were destructive for long-term mental health (Nydegger et al., 2011). The most reported adaptive coping strategy was talking with family, friends, and colleagues, whereas the most reported maladaptive coping strategy was related to help-seeking stigma. Participant F&R_1 described coping with a traumatic experience involving a motorbike fatality, stating “I started CPR, then they took his helmet off, and said ‘just stop’ to me, because he had cerebrospinal fluid coming out of his ears, a traumatic brain injury. That is the most stressful thing because it went from 0 to 100 quickly, and it is not a motor vehicle accident anymore. I did think about it for a couple days afterwards, and I drank six beers really quickly when I go home that night.” Following our analysis of the emergency services personnel experiences, we reflected on the applicability of this theme into sport. Not only is the understanding of adaptive versus maladaptive coping likely to be similar, but the behaviours listed within each are likely to be very similar in their existence and impact—perhaps only different in frequency between occupations.

### Workplace experiences

The researchers derived the theme *Workplace Experiences* from participant narratives that reflected both meaningful and traumatic experiences, with two sub-themes labelled: (a) Positive Experiences and (b) Traumatic Experiences. Consistent with Pratt et al. (2019), positive workplace experiences shaped confidence, learning, pride, and enjoyment. Conversely, traumatic experiences were described as repeated incidents that were difficult to cope with and had adverse effects (Nydegger et al., 2011). As traumatic events are characteristic of the emergency services, it is essential to have effective coping strategies, adequate self-care, and support networks in place to maintain resilience. AMB_6 described a stressful incident, “There was a suicide, where a lady hung herself on her balcony. We could not resuscitate her, and her two kids and husband were in the house, then it became a crime scene. It was really stressful, and it was in the small hours of the morning, and we obviously were trying to shield the kids, minimise their exposure that is really important. But then we just got called away to another priority job, pretty much had to pack up and go.” As with other themes, we subsequently reflected on the possibility of occupational experiences being crucial determinants of resilience in elite sport, and concluded that a similar pattern would be possible, albeit with some key differences in the exact events that would fall into positive versus traumatic.

### Burnout

We organised the final theme *Burnout* into two sub-themes: (a) Risks and Mitigations of Burnout and (b) Bucket of Bad Experiences. The latter of these represents a relatively unique theme that we felt needed to be captured and conveyed in this study

#### Risks and mitigations of burnout

Participants described excessive workload, stress, paperwork, and work schedules as causes for burnout. Regarding mitigating burnout, participants reflected emotional awareness and rest as critical resources for coping with occupational demands. Participants reported self-awareness and reflection were vital, and suggested flexible work arrangements, time off, and psychological services as mitigations their organisation could implement (e.g., AMB_6 reflected, “I think part of that is increasing self-awareness and understanding what is going to affect you and when it’s affecting you having the capacity to recognise what would contribute to reducing and mitigating burnout”).

#### Bucket of bad experiences

Three participants used the metaphor of a “bucket of bad experiences” when discussing burnout. Several participants described the belief that resilience to stress had a limit, burnout was inevitable and when it was going to occur depended upon how many negative experiences they had endured. F&R_2 described, “My bucket of bad experiences is small, compared to a guy that’s been working for 30 years and it just takes that one more job to tip him over.” As with several of the above themes, we subsequently mapped the contents of this theme against elite sport, concluding that (a) while risks and mitigations (or protective factors) may be analogised across both settings, they would likely contain different variables and events; and (b) the particular belief in a “bucket” that fills up appeared—to us at least—to be relatively unique to the emergency services personnel we spoke to.

## Discussion

The purpose of this study was to explore the definitions, meaning, and experiences of emotional awareness and resilience in emergency services personnel, and to better understand how these concepts relate with one-another. From there, we sought to extract lessons and insights for understanding resilience in elite sport. We studied 11 emergency services personnel, explored their meaningful experiences, and organised their narratives into seven interlinked themes. Our findings build upon research examining emotional awareness and add value to the literature on resilience in these specific populations (Hurley et al., 2020). We also make a noteworthy contribution to other qualitative research examining resilience in similar populations (O'Dowd et al., 2018; Lyu et al., 2020). Our findings illustrate how emergency services personnel define emotional awareness and resilience, and what it means to them in the context of their work. We also provide insight into their first-hand experiences in the field, how they cope with the daily challenges and traumas they face, as well as evidence of influential systemic workplace cultures. Furthermore, because of the identified similarities between emergency services personnel and elite athletes, our findings may have implications for elite athletes and research exploring their psychological resilience, well-being, and career success.

In line with the developing understanding of how to build and manage resilience (described earlier), our results show that emotional awareness and resilience were both closely connected to each other, and interlinked with many other themes. This rich connectivity and dense integration of concepts suggests complexity, and complex systems may be a better way of understanding the phenomena than simple linear associations (see Eagle and Pentland, 2005; Holland, 2006).

Resilience was defined by some participants as the ability to “bounce back” from stress, a common yet simplistic definition. However, participants more frequently spoke about resilience as being a rigid ability to withstand against stress, to just keep going no matter what and remain unemotional and unaffected by difficult experiences. This understanding differed from literature—particular the literature in sport—which defines resilience as dynamically adapting to difficult experiences through flexible coping (Woods, 2017). Participants also spoke about resilience as being crucial for coping with distressed members of the public, consistent with Kapoulitsas and Corcoran (2015). When comparing across emergency services vs. elite sport, we reflected that clarifying the evidence-based meaning and “mental models” of resilience—as being flexible and dynamic rather than fixed and rigid—may be just as beneficial in elite sport as it would appear to be for the emergency services personnel in our study.

The findings emphasised the importance of emotional awareness for effective coping, for resilience, to mitigate burnout and as a protective factor in the context of participants’ workplace environments. Emotional Awareness was a dominant theme throughout the narratives. Participants distinguished between interpersonal versus intrapersonal emotional awareness, both predictors of emotional intelligence (Maraichelvi and Rajan, 2013; Praditsang et al., 2015). Participants understood emotional awareness as the ability to notice, express, and understand one’s emotions (intrapersonal emotional awareness), a definition consistent with the literature (Armstrong et al., 2011). However, it could be argued that this is a simplistic understanding of emotional awareness. Lane and Smith (2021) outlined the complex levels of emotional awareness and how it influences significant change in psychotherapy to achieve positive mental health outcomes. Some participant narratives described emotional awareness as the ability to understand emotions and identify triggers, reactions, and responses, consistent with other findings (Williams et al., 2010; Mehling et al., 2012). Some participants also articulated that emotional awareness promoted effective social communication (interpersonal emotional awareness). This finding contributes to literature exploring the benefit of emotional awareness in social and cultural interactions (Ozcelik and Paprika, 2010) and the utility emotional awareness has in promoting interpersonal effectiveness and competence (Troth et al., 2012). Furthermore, the findings are congruent with Coholic (2011), who found that cultivating emotional awareness through mindfulness improved resilience. Consistent with Berking et al. (2010), Prati and Pietrantoni (2010), and Spratling et al. (2019), some participants specified that emotional awareness enhanced resilience, and directed the implementation of coping strategies. However, when exploring the narratives in this study, participants did not explore the nuances of emotional awareness. For example, participants did not differentiate between noticing an emotional trigger, versus noticing an emotional trigger then taking steps to self-regulate to reduce one’s emotional reactivity and engage in effective coping. These findings are novel and are worth expanding on in the literature. We reflected that education around the role of emotional awareness and training in how to draw upon this capability when dealing with stress would similarly benefit both emergency services personnel and elite athletes, similarly.

Notwithstanding, this study adds value beyond the existing literature, as it provides meaningful insights into the beliefs systems and cultures of each emergency service crew and what factors may interfere with the resilience and emotional awareness of their personnel (e.g., stigma, toxic masculinity beliefs, fears around vulnerability, and help-seeking). As above, these insights gained in a different high-stakes high-performance setting seem equally applicable in sport, and provide reinforcement that failing to address such beliefs may undermine initiatives designed to promote resilience in high performers. We explored beliefs and barriers that may shed light on why burnout and trauma are still a major concern in the emergency services. Although many participants in this study were cognisant of the benefits of emotional awareness, they did not actively engage in any practices to improve their own emotional awareness. We reflected based on this insight, that it may be interesting to explore the extent to which elite sport athletes and coaches engage is such practices. In this study, participants spoke about not feeling equipped to deal with traumatic situations they encountered and the risk this has for career longevity. For example, one participant reflected the training they did received “ticked the box”; however, was not sufficient in supporting officers in coping with traumatic stressors across their career. Furthermore, some police officers spoke about organisational beliefs interfering with their receptiveness to the mental health training they received, stating “civilians do not understand what we go through.” Most support staff working in elite sport will have experienced a similar message: “You just do not understand elite sport.”

In future, ethnographic research would provide additional rich data by observing emergency services personnel in their real-life work environment to gain a greater understanding of their experiences of resilience and emotional awareness. Moreover, longitudinal research would provide clarity on how workplace cultures and beliefs about resilience develop from recruitment to retirement in emergency service personnel. This provokes questions around whether and how new training programs can create systemic changes in the emergency services.

In the context of workplace stressors, participants reported needing resilience and emotional awareness to cope with the high volume of stressors, including dealing with not only distressed members of the public, but also to manage their own distress and potential trauma. This is consistent with other research highlighting the importance of emotional awareness (Williams et al., 2010) and resilience (Ogińska-Bulik and Zadworna-Cieślak, 2018) for coping with stress in emergency services personnel. Researchers have also established that maladaptive coping, alcohol use, and PTSD were related to lower levels of resilience and self-awareness in firefighters (Tomaka et al., 2017; Smith et al., 2018). Nonetheless, some participants in this study were not aware of the connection between maladaptive coping and reduced resilience, demonstrating limited awareness. Some also held the belief that burnout was inevitable, and it would occur when one’s ‘bucket of bad experiences’ overflowed. Collectively, these findings suggest a resilience paradox, whereby participants’ beliefs about resilience and burnout seem to impact their ability to maintain resilience. This paradox will likely impact the mental health and well-being of emergency services personnel over the course of their career. We did not anticipate this belief as being quite so prominent in elite sport, but would suggest elite sport practitioners may wish to check if it exists in settings they enter, due to the apparent impact it had in undermining more proactive coping: it reminded us as researchers of something akin to learned helplessness (Maier and Seligman, 1976).

Participants generally demonstrated awareness of mental health stigma and related attitudes towards help-seeking in their workplace culture (Karaffa and Koch, 2016). Similar to work by Hansson and Markström (2014) and Moffitt et al. (2014), potentially harmful beliefs about masculinity, coping and toughness were also apparent. However, despite their awareness of these attitudes, the majority of participants reported not feeling comfortable, not feeling the need, or not recognising the benefit of psychological help-seeking, and therefore had not sought a psychological service in the context of their work. This dialectic stimulates questions around how to effectively educate emergency services personnel about mental health and the preventative benefits of help-seeking in a method that they are receptive to, and create an environment where employees feel comfortable asking for help. Although there is evidence of cultural movements towards implementing preventative measures to promote mental health and subsequently improve resilience, the results of this study highlight that stigma surrounding psychological help-seeking exists. Nevertheless, it appears critical for the health and well-being of those in high-stakes high-performance settings to engage in preventative measures that cultivate emotional awareness, such as mindfulness, debriefing, seeking support, and reflective practices, to ensure their resilience is maintained across their career (Stanley et al., 2016).

These findings have evident implications for future emergency services personnel, but also for elite athletes. Research indicates that the careers of many successful athletes end prematurely due to psychological factors such as stress, low motivation, high anxiety, and self-confidence problems—all of which impact their resilience (Hill et al., 2010; Bicalho and Costa, 2018; Aron et al., 2019; Ozdemir, 2019; Daumiller et al., 2021). Other research found that low emotional intelligence was related to anxiety and self-confidence issues (Trigueros et al., 2019). Moreover, elite athletes are reported to have difficulty implementing adaptive coping mechanisms to manage psychological factors such as stress (Hill et al., 2010; Ozdemir, 2019). Qualitative research by Gulliver et al. (2012) and De Lenardo and Terrion (2014) examined mental health and help-seeking in elite athletes and found alarming patterns of help-seeking stigma, with barriers, including discrimination, low emotional literacy, organisational culture, and a lack of mental health awareness—mirroring the findings in the present study. As such, it may be worth exploring whether emotional awareness is a key factor in promoting athletes’ ability to cope with stress, engage in adaptive coping mechanisms (such as help-seeking), and promote their overall resilience and career success.

The results of this study provide a unique contribution to the research examining emotional awareness and resilience in the emergency services, as well as having implications for athletes and sport psychology research. While focussing on characterising the unique and meaningful experiences of emergency services personnel, we have identified potential discrepancies between their views of what it means to be resilient as set against the literature—a heavily sport-centric literature. In this way, the present study illustrates that resilience is construed and experienced in diverse ways, and this understanding may help us to plan, monitor, and adjust programs designed for elite sport (as well as emergency services personnel). In this study we have identified (or reinforced, at least) that there can be systemic/cultural beliefs and norms that interfere with the effectiveness of resilience training and mental health training programs. Thus, when it comes to real-world implementation in these high-stakes high performance settings, change must be strategically tailored to individual, team, and system levels (Barratt et al., 2018). Further, the findings in this study guide future research to examine similar patterns of occupational stress, coping and resilience in other high stress occupations such as elite athletes. If research can find meaningful ways to utilise emotional awareness to support the mental health and psychological well-being of personnel in these high stress occupations, it will likely promote organisational and career success, and reduce the incidence of burnout, PTSD, and significant mental health concerns on a global level. Septically, if practitioners or researchers identified similar beliefs to the ‘bucket’ metaphor detailed in this study, then a clear impediment to progress is present that needs to be addressed if program content is going to be implemented and effective. If performers truly believe that they are simply waiting for their bucket to fill, or their time to be ‘up’, then why would they learn and enact strategies for promoting dynamic, flexible resilience: such as emotional awareness and reflective practice?

### Limitations

Finite access to resources and time limited our ability to conduct further interviews and achieve stronger representation of the themes present in the narratives. Further limitations might include some participants’ lack of self-awareness, inaccuracy in self-evaluation and/or defensiveness, which may have limited findings to an incomplete map the relationship between emotional awareness and resilience. The study also recruited a sample of predominantly male participants from an upper socio-economic status in the ACT. Thus, the findings are unique, which – consistent with the constructivist approach adopted - may limit translatability to other regions, countries, and cultures. Nonetheless, the available data do identify coherent and meaningful insights that may inform future research. This qualitative study also has potential value beyond its contextual limits (Smith, 2018). This research has transferability, or inferential generalisability, through its meaningful topics, in-depth exploration of individual narratives, and rich theoretical expressions of reality that help us connect with our own experiences of remaining resilient and self-aware in the face of stress and trauma (Smith, 2018).

## Conclusion

This study explored emotional awareness and resilience, through the narratives and lived experiences of emergency services personnel. We worked with this group to generate benefits both for those in emergency services work, but also to generate additional insights that may benefit elite athletes. Most apparent, in our reflection on the study, was the critical need for emotional awareness and resilience for managing high-stakes challenges requiring exceptional skills and competence. We felt that this utility was highly transferrable between emergency services and elite sport. As such, we propose that the findings offer helpful insights, reinforcement, and perspective/framing to those promoting resilience and sustained high-performance in elite sport. As more evidence emerges supporting the important role emotional awareness plays in promoting resilience, the more likely organisations will be open to exploring how they can shift organisational cultures and effectively implement preventative measures to cultivate a more resilient emergency services workforce and elite athlete population. Moreover, this study evidences the need for correcting misperceptions about what it means to be resilient, and change stigmatised cultural beliefs about mental health and psychological help-seeking. This foundational, enabling step could ‘unlock’ the potential of interventions design to reduce the incidence of burnout, on a national, and potentially international level, and across multiple contexts. Effective and resilient workforces and organisations will accrue significant economic benefits to high stress occupations and the economy as a whole.

## Data availability statement

The datasets presented in this article are not readily available because the consent form signed by participants did not ask for permission for open sharing of the interview transcripts. As such, in order to share any data, we would need to seek fresh and specific consent from each participant. Requests to access the datasets should be directed to EJ.

## Ethics statement

The studies involving human participants were reviewed and approved by University of Canberra Human Research Ethics Committee. The patients/participants provided their written informed consent to participate in this study. Written informed consent was obtained from the individual(s) for the publication of any potentially identifiable images or data included in this article.

## Author contributions

EJ conducted all data collection, analysis, and manuscript drafting. RK supported the planning and design of the study, the data analysis and interpretation, and reviewing manuscript drafts. RK redrafted the article in response to reviewer feedback and handled final production queries. All authors contributed to the article and approved the submitted version.

## Conflict of interest

The authors declare that the research was conducted in the absence of any commercial or financial relationships that could be construed as a potential conflict of interest.

## Publisher’s note

All claims expressed in this article are solely those of the authors and do not necessarily represent those of their affiliated organizations, or those of the publisher, the editors and the reviewers. Any product that may be evaluated in this article, or claim that may be made by its manufacturer, is not guaranteed or endorsed by the publisher.
