# Supplementary material for: Sustaining optimal performance when the stakes could not be higher: Emotional awareness and resilience in emergency service personnel (with learnings for elite sport)
Source: Front Psychol. 2022 Aug 30;13:891585. doi: 10.3389/fpsyg.2022.891585 (PMC9472212; doi:10.3389/fpsyg.2022.891585)
Supplement: Supplementary file 1 [file Table_1.docx]

Supplementary Table 1

*Adaptive and Maladaptive Coping Strategies*

| Adaptive Coping | Maladaptive Coping |
| --- | --- |
| Debriefing, talking and social support | Would not seek help/did not seek help |
| Reflection, processing and acceptance | Active avoidance |
| Problem-solving and planning | Alcohol and drug use |
| Hobbies and activities | Problematic cognitions |
| Professional help-seeking | Getting angry or frustrated |
| Meditation, mindfulness and spirituality | No conscious effort to improve coping |
| Keeping busy and distraction | Binge eating/emotional eating |
| Taking time off and down time | Poor self-care |
| Expressing emotion e.g., crying | Panic |
| Self-talk | Gambling |
| Breathing | Obsessive behaviours |
| Gratitude journal | Other impulsive behaviour |
| Humour | Suicidal ideation |
| Diet and nutrition | Using social media |
| Sleep | Social isolation |
